# Dissecting the Molecular Interactions between Wheat and the Fungal Pathogen *Zymoseptoria tritici*

**DOI:** 10.3389/fpls.2016.00508

**Published:** 2016-04-15

**Authors:** Graeme J. Kettles, Kostya Kanyuka

**Affiliations:** Department of Plant Biology and Crop Science, Rothamsted ResearchHarpenden, UK

**Keywords:** *Septoria tritici* blotch (STB), *Mycosphaerella graminicola*, effector, pathogen-associated molecular pattern (PAMP), chitin, disease resistance, PAMP-triggered immunity (PTI), wheat

## Abstract

The Dothideomycete fungus *Zymoseptoria tritici* (previously known as *Mycosphaerella graminicola* and *Septoria tritici*) is the causative agent of *Septoria tritici* leaf blotch (STB) disease of wheat (*Triticum aestivum* L.). In Europe, STB is the most economically damaging disease of wheat, with an estimated ∼€1 billion per year in fungicide expenditure directed toward its control. Here, an overview of our current understanding of the molecular events that occur during *Z. tritici* infection of wheat leaves is presented. On the host side, this includes the contribution of (1) the pathogen-associated molecular pattern-triggered immunity (PTI) layer of plant defense, and (2) major *Stb* loci for resistance against *Z. tritici*. On the pathogen side of the interaction, we consolidate evidence from recent bioinformatic, transcriptomic and proteomic studies that begin to explain the contribution of *Z. tritici* effector proteins to the biphasic lifestyle of the fungus. This includes the discovery of chitin-binding proteins in the *Z. tritici* secretome, which contribute to evasion of immune surveillance by this pathogen, and the possible existence of ‘necrotrophic’ effectors from *Z. tritici*, which may actively stimulate host recognition in a manner similar to related necrotrophic fungal pathogens. We finish by speculating on how some of these recent fundamental discoveries might be harnessed to help improve resistance to STB in the world’s second largest food crop.

## The *Zymoseptoria tritici* Life Cycle and Pathogenesis Program

*Zymoseptoria tritici* is an ascomycete fungus belonging to the family Mycosphaerellaceae in the class Dothideomycetes. The *Z. tritici* lifestyle is described as hemibiotrophic, with two distinct phases of infection. Following inoculation onto the leaf surface by rain splash, spores germinate and the fungus invades exclusively through the stomata before undergoing a prolonged asymptomatic phase of very slow hyphal growth in the apoplastic space between mesophyll cells. There is no evidence of host cell penetration or formation of specialized feeding structures such as haustoria (Kema et al., 1996). This phase has also often been refered to as ‘biotrophic.’ Recent transcriptomic and metabolic profiling of *Z. tritici* infection of susceptible wheat, however, indicated that the fungus’ own lipids and fatty acids are most likely used as the main energy sources during this phase. That said, a number of fungal genes encoding secreted cutinases and lipases were found to be significantly up-regulated during the asymptomatic phase, and it has been proposed that host lipids (e.g., leaf surface waxes) may also be utilized (Rudd et al., 2015). This first infection phase is also characterized by the absence, or very weak defense response (Keon et al., 2007; Yang et al., 2013b; Rudd et al., 2015) suggesting that *Z. tritici* is able to successfully suppress or avoid immune elicitation. However, the ratio of fungal to wheat biomass at this stage is extremely low and therefore any local host defense response (if present) may be diluted out and be undetectable by current methods. The asymptomatic phase typically lasts 7–10 days following inoculation depending on the particular cultivar-isolate combination, after which there is a rapid transition to the symptomatic phase, which is frequently referred to as ‘necrotrophic.’ This second infection phase is typified by the large-scale reprogramming of both host and pathogen transcriptomes, a strong activation of host defense responses culminating in apoptotic-like rather than necrotic cell death and release of nutrients into the leaf apoplast and, as a consequence, a substantial build-up of fungal biomass (Keon et al., 2007; Yang et al., 2013b; Rudd et al., 2015). Macroscopically visible dead leaf areas then expand to form irregularly-shaped blotches (lesions) in which fungal asexual sporulation structures called pycnidia develop. These erupt through stomatal openings and release pycnidiospores that may initiate further rounds of infection when spread to healthy tissues via rain splash.

## *Z. tritici* Effectors and Host Cell Surface Immune Receptors Play Key Roles During the Asymptomatic Infection Phase

The principle function of the immune system is to recognize non-self molecules that betray the presence of an invader. Non-self molecules that trigger innate immune responses are considered pathogen-associated molecular patterns (PAMPs) (Jones and Dangl, 2006). Several fungal PAMPs have been described, including chitin (polymer of long chains of *N*-acetylglucosamine), β-glucans (polymers of glucose), mannans (polymers derived from mannose, galactose, and glucose) derived from cell walls, and ergosterol found in cell membranes (Klarzynski et al., 2000; Lochman and Mikes, 2006; McGreal et al., 2006; Shetty et al., 2009; Sánchez-Vallet et al., 2014). Successful immune activation following PAMP elicitation leads to PAMP-triggered immunity (PTI) (Jones and Dangl, 2006).

**FIGURE 1:**
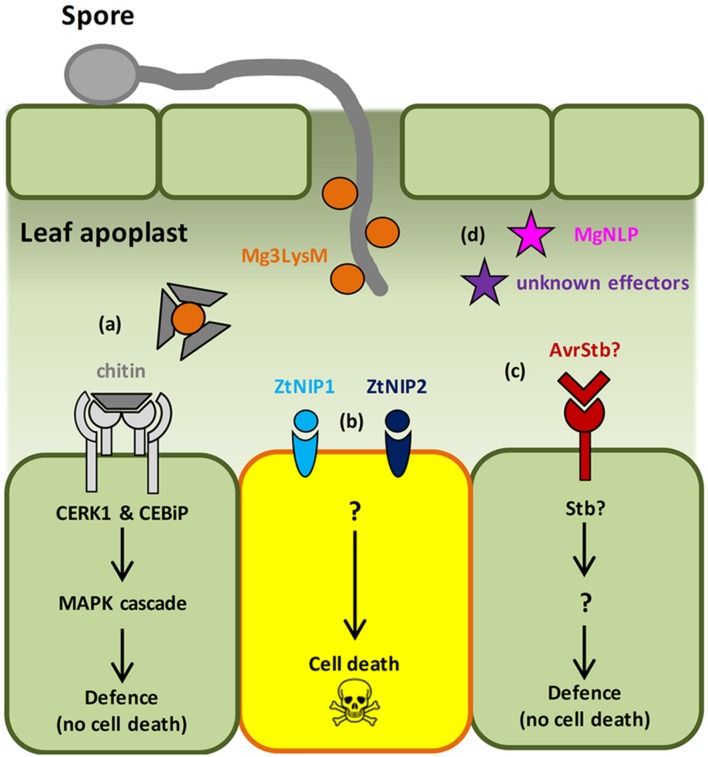
**Molecular events in the *Zymoseptoria tritici*–wheat interaction.** **(a)** Fungal PAMP chitin is recognized by the host receptors Chitin Elicitor Binding Protein (CEBiP) and Chitin Elicitor Receptor Kinase 1 (CERK1), triggering MAP kinase cascades and immune activation. Multi-functional LysM-domain containing effector Mg3LysM scavenges chitin to suppress immunity and protects fungal hyphae from wheat chitinases. **(b)** ‘Necrotrophic’ effectors, Necrosis-Inducing Protein 1/2 (ZtNIP1/2), induce host cell death via an unknown mechanism. **(c)** *Stb* gene-specified resistance presumably triggered following recognition of cognate fungal effectors (AvrStb) secreted into the apoplast. This results in arrest of pathogen growth via an unknown mechanism that does not involve HR. **(d)** The Necrosis and Ethylene-Inducing Peptide 1 (NEP1)-like effector protein MgNLP has an unknown function(s) during wheat infection, but triggers cell death in dicotyledonous plants. Unknown effectors – *Z. tritici* effectors predicted from bioinformatic analyses.

For a successful pathogen to usurp host immunity, the invader must deploy secreted effector proteins, metabolites or other mechanisms to overcome host defenses triggered during PTI. Of particular importance to fungal-plant interactions (including the *Z. tritici –* wheat interaction) is the PAMP chitin, which represents one of the major components of fungal cell walls and is naturally absent in plants (Sánchez-Vallet et al., 2014). Chitin functions as an elicitor of defense in both dicots and monocots, including a number of model and crop species, such as rice and wheat. Preventing or suppressing chitin-triggered defense is therefore likely to be important for any fungal pathogen. The Ecp6 effector from the fungus *Cladosporium fulvum* that causes the tomato leaf mold disease (Bolton et al., 2008) was demonstrated to have ultrahigh chitin-binding affinity via cooperation of two out of its three lysin motifs (LysM) (de Jonge et al., 2010). *Z. tritici* encodes three LysM-containing proteins (Mg1LysM, Mg3LysM, and MgxLysM) with Mg3LysM being the functional ortholog of *C. fulvum* Ecp6. It has been recently demonstrated that both Mg1LysM and Mg3LysM are transcriptionally highly up-regulated during symptomless colonization of wheat and both bind chitin (Marshall et al., 2011). However, only Mg3LysM is capable of blocking chitin-induced elicitation of wheat defenses (**Figure 1**). Curiously, both Mg3LysM and Mg1LysM also have a unique protective effect for *Z. tritici*, shielding the fungal cell wall from digestion by host chitinases (Marshall et al., 2011). This activity has not been observed for LysM effectors from other fungi including Ecp6. These results demonstrate the importance of chitin-binding effectors during the early stage of plant colonization.

Molecular recognition of chitin fragments in cereals requires the Chitin Elicitor Binding Protein (CEBiP) and the Chitin Elicitor Receptor Kinase 1 (CERK1) (Kaku et al., 2006; Shimizu et al., 2010). CEBiP was originally identified as a chitin receptor in rice (OsCEBiP) (Kaku et al., 2006) and is a membrane-anchored extracellular protein that is predicted to be heavily glycosylated (Kaku et al., 2006). The nature and importance of these glycosylations to OsCEBiP function are currently unknown. OsCEBiP recognizes and binds chitin via its extracellular LysM-containing domain but contains no intracellular signaling domain, and is therefore unable to initiate immune signaling alone. OsCERK1 is a transmembrane protein with an extracellular LysM-containing domain and an intracellular kinase domain (Shimizu et al., 2010). OsCERK1 is essential for the generation of a Mitogen-Activated Protein (MAP) kinase signaling cascade following chitin elicitation (Shimizu et al., 2010). The precise mechanism of chitin binding by OsCEBiP has recently been elucidated and shown to require receptor dimerization (Hayafune et al., 2014).

Wheat contains one *CEBiP* and two sequence-related *CERK1* genes that show high degrees of similarity to their rice orthologs (81 and 86%, respectively) (Lee et al., 2014). Whilst wheat *CEBiP* and *CERK1* are understudied relative to their rice counterparts, their importance during the *Z. tritici* asymptomatic infection phase have been revealed in a recent study (Lee et al., 2014). It was demonstrated that the *Z. tritici* mutant lacking a functional Mg3LysM gene, which normally shows dramatically reduced virulence, was able to regain nearly wild-type virulence on wheat plants in which either *CEBiP* or *CERK1* was silenced through Virus-Induced Gene Silencing (VIGS) (Lee et al., 2014, 2015). This is consistent with the role of the Mg3LysM effector in chitin sequestration (Marshall et al., 2011).

## *Z. tritici* Effectors Potentially Contributing to the Second, Symptomatic Infection Phase

Bioinformatics-based genome-wide approaches have identified several hundred *Z. tritici* genes encoding candidate secreted proteins that bear hallmarks of effectors identified from other pathosystems, i.e., small, cysteine rich, and expressed *in planta* (do Amaral et al., 2012; Gohari et al., 2015). Approximately 100 of these candidate effector genes have been shown to peak in expression either during the switch from asymptomatic to symptomatic infection phase, or during the symptomatic phase (Yang et al., 2013b; Rudd et al., 2015). Some of these effectors also become quite abundant during this second phase and can be detected in apoplastic fluids from infected leaf tissues using proteomics analysis (Yang et al., 2013a).

One of the *Z. tritici* effectors transcriptionally upregulated toward the late stage of symptomless colonization by *Z. tritici* is MgNLP, a member of the Necrosis and Ethylene-inducing Peptide 1 (NEP1)-like (NLP) protein family identified and characterized from a number of bacteria, fungi and oomycetes, including many plant pathogens (Gijzen and Nürnberger, 2006). Whilst NLPs often occur in large families, thus making functional redundancy problematic, *Z. tritici* harbors only a single NEP1-like protein (Motteram et al., 2009). Similar to other NLPs, MgNLP was found to induce defense-related responses and cell death in leaves of dicotyledonous plants (Motteram et al., 2009). Curiously, MgNLP does not induce defense genes or trigger cell death in wheat, and a targeted *MgNLP* deletion mutant of *Z. tritici* is not compromised in its ability to infect wheat (Motteram et al., 2009). Therefore, MgNLP may be functionally redundant and its exact function during wheat infection remains elusive (**Figure 1**).

It has been recently shown that liquid *Z. tritici* culture filtrates contain proteinaceous effectors able to induce cell death when injected into wheat leaves (M’Barek et al., 2015). This study identified two putative ‘Necrosis-Inducing Proteins’ designated as ZtNIP1 and ZtNIP2 through fast protein liquid chromatography and liquid-chromatography mass spectrometry fractionation of culture filtrates. Syringe-infiltration of heterologously expressed ZtNIP1 and ZtNIP2 into wheat leaves induced cell death in a cultivar-dependent fashion (M’Barek et al., 2015). Whilst the specific cell death-inducing activity of these proteins is as yet unknown, these findings point to the existence of interactions between the *Z. tritici* ‘necrotrophic’ effectors and the corresponding host susceptibility factors (**Figure 1**), similar to those described for necrotrophic pathogens which infect wheat, *Phaeosphaeria nodorum* (syn. *Stagonospora nodorum*) and *Pyrenophora tritici-repentis* (Tan et al., 2015).

Functional characterisation of other candidate phase-specific *Z. tritici* effectors identified through bioinformatic, proteomic and genome-wide transcriptomic analyses (do Amaral et al., 2012; Gohari et al., 2015; Rudd et al., 2015) is currently underway in several laboratories around the globe including ours, and appears a rich source of future discoveries.

## Managing *Septoria tritici* Blotch Disease in Wheat

The control of STB disease in wheat crops currently relies on the intensive use of fungicides with mixtures of azole and succinate dehydrogenase inhibitor (SDHI) fungicides being most effective (Torriani et al., 2015). Resistance to STB is also an important target in wheat breeding, recently catalyzed by new EU regulations restricting the use of some of the most potent fungicide products as well as increasingly frequent reports of fungicide resistance in field populations of *Z. tritici*^1^. In comprehensive worldwide wheat germplasm screening programs, a number of exotic as well as synthetic wheat genotypes have been identified as good sources of STB resistance. At least 20 distinct genetic loci have been identified (*Stb* loci) that confer qualitative, often isolate-specific resistance to *Z. tritici*. In addition, a large number of quantitative trait loci (QTL), which make smaller contributions to the *Z. tritici* resistance phenotype, have been mapped genetically. For a comprehensive up-to-date review of both *Stb* loci and resistance QTLs see Brown et al. (2015).

Although major STB resistance loci (designated as *Stb1* through to *Stb18*, *StbSm3*, and *StbWW*) have been identified in hexaploid wheat, all of these with the exception of *Stb16q* provide protection against individual or small groups of *Z. tritici* isolates. *Stb6* is one of the better-characterized resistance genes. *Stb6*-based resistance conforms to the gene-for-gene hypothesis, whereby avirulent *Z. tritici* isolates (such as the reference isolate IPO323 with fully sequenced genome; Goodwin et al., 2011) carry the matching *AvrStb6* gene (Brading et al., 2002). However, the identity of *AvrStb6* is not yet known and so the frequency of this avirulence gene in current *Z. tritici* field populations remains to be determined. Interestingly, *Stb6* is found in many commercial wheat cultivars and breeding lines originating from Europe, China, Israel, and the United States (US) that are known sources of *Z. tritici* resistance (Chartrain et al., 2005). This gene appears to contribute some resistance to STB in the field at least in the United Kingdom (UK) (Arraiano et al., 2009) indirectly suggesting that *AvrStb6* may be relatively well represented in regional field populations of *Z. tritici*. Frequencies of avirulence toward other known *Stb* genes in field populations of the fungus are unknown. However, it is plausible that these may be sufficiently high for some individual *Stb* genes to provide a certain degree of resistance durability. With this in mind, it is worth noting that US wheat breeders have been actively deploying two major resistance genes, *Stb1* and *Stb4*, since the early 1970s (Goodwin, 2007). *Stb1* provided long-lasting resistance to wheat in the central USA (Adhikari et al., 2004), and *Stb4* remained effective in California for about 15 years until the effectiveness of this gene decreased (Jackson et al., 2000). Efficacy and durability of the other known *Stb* genes against natural fungal populations is not known. Importantly, some wheat genotypes used as major sources of resistance to STB in current world breeding programs, for example, TE9111, Kavkaz-K4500 L.6.A.4 and Veranopolis, all contain ≥3 *Stb* genes (Chartrain et al., 2004; Kollers et al., 2013). This strongly suggests that pyramiding *Z. tritici* isolate-specific resistance genes may be an effective strategy for developing wheat cultivars with high levels of field resistance.

*Stb16q* is particularly interesting from the disease resistance breeding point of view because this gene appears to confer broad-spectrum seedling stage resistance, as so far no resistance-breaking *Z. tritici* isolates have been found. *Stb16q* has been identified in synthetic hexaploid wheat and was shown to originate from one of its parents, the diploid wild wheat species *Aegilops tauschii* (Tabib Ghaffary et al., 2012). Interestingly, broad-spectrum resistance to *Z. tritici* has also been identified in the diploid wheat *Triticum monococcum* and was shown to be controlled by a single genetic locus *TmStb1* (Jing et al., 2008). Efforts are currently underway to introgress *TmStb1* into hexaploid wheat^2^. It would be interesting to evaluate the usefulness of *Stb16q* and *TmStb1* for potentially achieving durable broad-spectrum resistance to *Z. tritici* in bread wheat as well as to explore further the full potential of synthetic hexaploid wheat and wild relatives of wheat as novel sources of resistance to *Z. tritici.*

The functional characterisation of several of the known *Stb* genes would greatly increase fundamental understanding of the wheat immune system and may facilitate the identification of novel candidate resistance genes using bioinformatic approaches.

## Future Strategies For STB Disease Resistance

### Exploiting PTI to Enhance Resistance to *Z. tritici*

The battle that exists in the apoplastic space for chitin binding goes some way to determining the overall outcome of the *Z. tritici*-wheat interaction. The comparative chitin-binding affinity of the wheat receptors CEBiP and CERK1 relative to chitin-scavenging ability of the fungal effector Mg3LysM is unknown. It is not unreasonable to assume that pathogen effectors such as Mg3LysM, under high evolutionary pressure, might be optimized for chitin binding affinity. One novel strategy, therefore, would be to engineer wheat immune receptors to match or exceed the chitin affinity of the competing fungal effector proteins. Such a tactic might allow ‘supercharging’ the PTI layer of the wheat immune system. Indeed, such a resource might already exist in nature. However, no study has yet examined the variation in chitin sensitivity across commercial wheat cultivars or their wild relatives.

Furthermore, chitin is likely not the only PAMP present in the apoplastic space during *Z. tritici* infection. β-glucans are another major components of the fungal cell wall and preparations of these compounds from *Z. tritici* have been shown to elicit defense responses in wheat and to confer some resistance to a normally susceptible cultivar (Shetty et al., 2009). Understanding of how plants perceive β-glucans is immature in comparison to perception of chitin. Indeed, it is not known if a dedicated membrane-bound immune receptor exists. Nonetheless, enhancement of multiple immune receptors concurrently might offer the same benefits as has been widely discussed with the stacking of dominant isolate- or race-specific resistance (*R*) genes.

### Exploiting Major Resistance Genes for Enhanced Resistance to *Z. tritici*

Whilst considerable scientific and breeding efforts have been directed toward the identification of useful sources of *Z. tritici* resistance and development of elite resistant wheat, fully resistant varieties are not yet available to farmers. Taking the UK as an example, the Agriculture and Horticulture Development Board (AHDB) 2016/17 recommended list for winter wheat provides information on the level of disease resistance for current commercially available wheat cultivars^3^. For *Z. tritici* resistance, the vast majority of cultivars (34 of 36) score 6 or less, on a scale of 0–9 (whereby 0 indicates high level of susceptibility and 9 indicates high level of resistance). Only two cultivars (Graham and KWS Siskin) scored as high as 7. This highlights the need for development of novel strategies in breeding for *Z. tritici* resistance.

Single major *Stb* genes can confer complete resistance to *Z. tritici*, albeit against a relatively narrow range of isolates harboring the corresponding avirulence (*Avr*) genes (**Figure 1**). Deployment of single resistance genes rarely provides durable disease resistance as avirulent isolates of a target pathogen need only to escape this single recognition event in order to regain virulence. Stacking of multiple *Stb* genes against *Z. tritici* either through marker assited breeding or via cysgenics will likely provide more effective resistance as evidenced by the prevalence of multiple *Stb* genes in highly resistant germplasm. Biochemical and molecular characterisation of *Stb* gene products will aid the design of optimal stacking strategies and may also facilitate identification of novel *Stb* alleles.

### Effector-Assisted Screening for Novel Resistances in Wheat

For biotrophic pathogens, recognition of an effector by a corresponding major host resistance protein often leads to induction of the hypersensitive response (HR) and localized cell death that correlates with limited pathogen spread beyond the primary inoculated loci. By contrast, recognition of some ‘necrotrophic’ pathogen effectors triggers induction of host cell death that leads to host susceptibility (Faris et al., 2013). Notable examples are the toxic effectors from the pathogens *P. nodorum* and *P. tritici-repentis* (*Ptr*) that cause glume blotch and tan spot disease of wheat, respectively (Faris et al., 2013; Vleeshouwers and Oliver, 2014). For *Ptr* ToxA, effector sensitivity correlates with disease susceptibility across a collection of wheat cultivars (reviewed in Faris et al., 2013). ToxA sensitivity is dependent on the wheat gene *Tsn1*, although there is no evidence of a direct ToxA-Tsn1 interaction (Faris et al., 2010). Nonetheless, screening of Australian wheat germplasm using recombinant ToxA protein has helped direct breeding programs toward the elimination of *Ptr* susceptibility. Effector-directed breeding reduced the area of ToxA-sensitive wheat grown in Western Australia by nearly 50% over the first three growing seasons since the program was initiated in 2009 (Vleeshouwers and Oliver, 2014). Similar programs may be initiated to breed out susceptibility to *Z. tritici*. However, knowledge of *Z. tritici* susceptibility genes in wheat and identification of ‘necrotrophic’ effectors potentially contributing to disease is less advanced than for pathogens such as *Ptr*. Recent description of *Z. tritici* effectors ZtNIP1 and ZtNIP2 that induce cell death in some but not all wheat cultivars (M’Barek et al., 2015) suggest that sensitivity to ‘necrotrophic’ effectors may potentially contribute to *Z. tritici* susceptibility. If proven, this may be exploited for breeding resistant (or at least less susceptible) wheat in a similar fashion as discussed above for the wheat–*Ptr* interaction.

## Conclusion and Future Perspectives

Molecular characterisation of the major events occurring during *Z. tritici* infection of wheat has enabled more thorough understanding of disease progression. Significant progress has been made, for example, in describing the candidate *Z. tritici* effector repertoire. However, how the >100 candidate effectors act in concert to induce disease remains unknown. Likewise, the existence of *Stb* loci conferring strong isolate-specific resistance has been known for some time, but only one has so far been cloned or functionally characterized (unpublished). Nonetheless, progress that has been made give reason to be optimistic that application of more advanced (and rapid) molecular tools will allow fuller understanding of STB disease development, taking it on par with model pathogens.

Where perhaps a greater problem lies, however, is the translation of fundamental findings into real-world impact. But even here, trails have been blazed using other pathosystems that may point the way for *Z. tritici* research. The implementation of an effector-directed breeding program against *Ptr* (discussed above) is a prime example of how fundamental research can lead to low-cost, time-saving tools for the benefit of agriculture.

It is speculated that introduction of *Z. tritici* susceptibility into breeding programs came as a result of breeding for higher yields combined with tight association of gene loci conditioning susceptibility and those for yield potential (Torriani et al., 2015). In the event that culprit susceptibility gene loci are identified and characterized biochemically in wheat, genome editing technologies such as CRISPR-Cas9 (Wang et al., 2014) might be used in their modification or inactivation without the necessary yield loss penalties demanded by agriculture.

## Author Contributions

GK and KK jointly conceived this mini review. GK produced the first draft and GK and KK then critically revised the manuscript. Both, GK and KK approve the final version of the manuscript. Both authors agree to be accountable for all aspects of the manuscript in ensuring that questions related to the accuracy or integrity of any part of the work are appropriately investigated and resolved.

## Conflict of Interest Statement

The authors declare that the research was conducted in the absence of any commercial or financial relationships that could be construed as a potential conflict of interest.

## Footnotes

1 http://cereals.ahdb.org.uk/press/2015/december/16/statement-on-sdhi-fungicides-and-resistance-risk-in-cereals-issued-by-frag-uk.aspx

2 http://www.wgin.org.uk

3 http://cereals.ahdb.org.uk/varieties/ahdb-recommended-lists.aspx
